# The effects of normobaric hypoxia on the leukocyte responses to resistance exercise

**DOI:** 10.5114/biolsport.2023.112087

**Published:** 2022-01-03

**Authors:** Giselle Allsopp, Jackson Barnard, Samuel Goodear, Samantha Hoffmann, Garth Stephenson, Alex Addinsall, Craig Wright

**Affiliations:** 1 Institute for Physical Activity and Nutrition, School of Exercise and Nutrition Sciences, Deakin University, Geelong, Victoria, Australia; 2 Centre for Sport Research, School of Exercise and Nutrition Sciences, Deakin University, Geelong, Victoria, Australia; 3 School of Medicine, Deakin University, Geelong, Victoria, Australia; 4 Current address: Department of Physiology and Pharmacology, Karolinska Insitutet, 171 77 Stockholm, Sweden

**Keywords:** Strength training, Simulated altitude, White blood cells, Neutrophils, Immune

## Abstract

There is growing interest in the use of systemic hypoxia to improve the training adaptations to resistance exercise. Hypoxia is a well-known stimulator of the immune system, yet the leukocyte responses to this training modality remain uncharacterised. The current study characterised the acute leukocyte responses to resistance exercise in normobaric hypoxia. The single-blinded, randomised trial recruited 13 healthy males aged 18–35 years to perform a bout of resistance exercise in normobaric hypoxia (14.4% O_2_; n = 7) or normoxia (20.9% O_2_; n = 6). Participants completed 4 × 10 repetitions of lower and upper body exercises at 70% 1-repetition maximum. Oxygen saturation, rating of perceived exertion and heart rate were measured during the session. Venous blood was sampled before and up to 24 hours post-exercise to quantify blood lactate, glucose and leukocytes including neutrophils, lymphocytes, monocytes, eosinophils and basophils. Neutrophils were higher at 120 and 180 minutes post-exercise in hypoxia compared to normoxia (p<0.01), however lymphocytes, monocytes, eosinophils and basophils were unaffected by hypoxia. Oxygen saturation was significantly lower during the four exercises in hypoxia compared to normoxia (p < 0.001). However, there were no differences in blood lactate, heart rate, perceived exertion or blood glucose between groups. Hypoxia amplified neutrophils following resistance exercise, though all other leukocyte subsets were unaffected. Therefore, hypoxia does not appear to detrimentally affect the lymphocyte, monocyte, eosinophil or basophil responses to exercise.

## INTRODUCTION

Resistance exercise causes a profound change in the number of leukocytes in the systemic circulation that can persist for hours after the exercise bout. Although endurance exercise has featured in much of the exercise immunology literature, interest is growing in how the five leukocyte populations respond to different resistance training protocols; neutrophils, lymphocytes, monocytes, eosinophils and basophils [1].

An acute bout of moderate- to high-intensity resistance exercise increases the number of circulating neutrophils that typically peak between 90 and 120 minutes post-exercise [2, 3]. Similarly, monocyte concentrations increase immediately following a bout of resistance exercise and remain elevated for up to 120 minutes [2, 3]. In addition to their role in innate immunity, these phagocytic populations are important mediators of skeletal muscle repair and adaptation [4]. The mechanical strain associated with resistance exercise induces myofiber damage, which results in the release of inflammatory cytokines from skeletal muscle [5]. These inflammatory cytokines and other chemotactic factors attract neutrophils and macrophages to skeletal muscle [6], where they play a vital role in the degradation of damaged muscle and the subsequent adaptation of skeletal muscle tissue [4].

Lymphocytes are typically elevated 2-fold immediately following an acute bout of high-intensity resistance exercise, termed lymphocytosis [7]. Lymphocytopenia then occurs, where lymphocyte concentrations drop below resting levels approximately two hours post-exercise [7]. The subsequent lymphocytopenia in the hours following resistance exercise could be partly caused by the sequestering of lymphocytes back into tissues such as the lungs and lymphoid tissues, in addition to any damaged muscle tissue [8]. Although lymphocyte subsets such as T lymphocytes likely contribute to skeletal muscle regeneration [9], much of the exercise immunology literature focuses on their role in resistance to infection [10]. The mechanisms modulating the immune cell responses to exercise are not fully understood, though they likely include an increase in circulating adrenaline during exercise that stimulates β-2-adregernic receptors on neutrophils [11], lymphocytes [12] and macrophages [13, 14]. The sympathetic stimulation of adrenaline increases almost linearly with exercise intensity and therefore the leukocyte response is likely intensity dependent [14, 15]. Whilst the leukocyte responses to training variables such as nutrient status [16] and inter-set rest periods [2] are well characterised, there are many novel resistance training strategies that have not been investigated. There is growing interest in the use of systemic hypoxia to improve the training adaptations to resistance exercise [17], yet little is known about the collective effects of resistance exercise and systemic hypoxia on circulating leukocyte populations.

Performing resistance exercise at simulated altitude is a novel strategy to enhance muscle hypertrophy and strength gains to a greater extent than the equivalent training in normoxia [17, 18]. Many recreational training facilities now offer simulated altitude training rooms where the air pressure is equivalent to sea level, but the environmental O_2_ is reduced to 12–16%, equating to approximately 1,800–4,000 m above sea level (termed normobaric hypoxia) [19]. Although most hypoxic training studies focused on the muscle hypertrophy and strength adaptations, systemic hypoxia is a potent regulator of circulating leukocyte populations. Acute exposure to hypoxia for twenty minutes can modulate the number of circulating leukocytes such as lymphocytes (natural killer cells) and monocytes in healthy individuals [20]. Pedersen *et al.* [21] suggested that acute exercise and hypoxia exposure are additive stimuli to the im mune system, where greater immunological stress is induced by performing exercise in hypoxia. A contributor to this response may be an increase in relative exercise intensity, where there is increased reliance on anaerobic metabolism and increased sympathetic activity in hypoxia [22, 23]. However, Katayama *et al.* [24] showed that performing dynamic leg exercise in hypoxia (12.7% O_2_) elicits greater muscle sympathetic nerve activity than normoxia even when controlling for relative exercise intensity. Systemic hypoxia also activates other pathways including hypoxia-inducible factor 1 (HIF-1) and Nuclear Factor kappa-light-chain-enhancer of activated B cells (NFκB) which are both well-known to stimulate leukocyte populations [25, 26].

Acute resistance exercise alone can mobilise leukocytes into the circulation through increased circulating adrenaline, noradrenaline and growth hormone [27]. Hypoxia can amplify the acute hormonal responses to resistance exercise in young adults [28, 29] and thus hypoxia may also amplify the leukocyte responses to resistance exercise. Currently, there are no studies investigating the leukocyte responses of healthy adults to moderate-intensity resistance exercise in hypoxia.

Therefore, this study characterised the acute leukocyte responses of healthy adults to a single bout of resistance exercise in either normobaric hypoxia or normoxia. We hypothesised that hypoxia would amplify the acute leukocyte responses to resistance exercise.

## MATERIALS AND METHODS

### Experimental design

The study was designed to investigate the acute effects of hypoxic resistance exercise on the dependant variable; leukocytes. The study was performed in accordance with the ethical standards of the Helsinki Declaration and was approved by the Deakin University Human Research Ethics committee (reference number: 2016-308). After the nature and risks of the research were explained to the participants, written informed consent was obtained from participants.

Statistical power for the study was calculated using a priori in G*Power through repeated measures analysis of variance (ANOVA), within-between factors (the F test; Version 3.1.9.4; Universität Kiel, Germany). The power analysis was designed to examine the additive effect of hypoxia on the leukocyte response to resistance exercise. Therefore we identified a study that examined an additive stimulus (vitamin supplementation) on the leukocyte responses to resistance exercise [30]. Neutrophils were selected as the largest leukocyte subpopulation in systemic circulation and 120 minutes post-exercise was selected as the most meaningful time point. Cohens d was first calculated and then transformed into effect size f. The following parameters were used; Alpha error probability (0.05), power (1-β error probability; 0.80), groups (two), number of measurements (two; pre-exercise and 2 hours post-exercise), standard correlation among repeated measures (0.5), non-sphericity correction (one). Therefore, to detect an effect size (f) of 0.620 using a two-way repeated measures ANOVA for mean neutrophil count, a minimum of four participants in each group were required for this study. Thirteen participants were recruited to account for potential dropout and variation in leukocyte responses.

### Subjects

The single-blinded randomised study recruited 13 apparently healthy adult males aged 18–35 years. Participants were randomly allocated to complete a single bout of resistance exercise in normoxia at 20.93% O_2_ (n = 6) or hypoxia at 14.4% O_2_ (n = 7). Normobaric hypoxia was achieved in an environmental chamber using a normobaric hypoxic generator (Pulford Systems, Australia). Ambient air was enriched with N_2_ to reduce the O_2_ in the environmental chamber to 14.4% O_2_, equivalent to an altitude of approximately 3,300 m above sea level [19]. Participants were non-smokers, non-diabetic and recreationally active. Participants had not partaken in a heavy resistance training program for a minimum of six months prior to the study and had no prolonged exposure to altitude above 3000 m within the previous six months.

**TABLE 1:** Participant characteristics. Data is presented as mean ± SD.

	Hypoxia (n = 7)	Normoxia (n = 6)
Age (years)	22.1 ± 2.5	23.7 ± 2.6
Height (m)	1.81 ± 0.08	1.83 ± 0.03
Weight (kg)	75.9 ± 9.6	77.6 ± 9.5
BMI ([kg/m^-1^]^2^)	23.3 ± 2.5	23.3 ± 3.2
V O2max (mL.kg^-1^.min^-1^)	49.4 ± 2.96	47.7 ± 1.69

M, metres; kg, kilograms; BMI, body mass index.

### Procedures

Participants attended Deakin University Waurn Ponds for anthropo-metric measurements and a familiarisation to the exercise testing protocols. After at least three days’ rest, participants returned for physical fitness testing in normoxia at approximately 7.30 am in a fasted and rested state. Participants were asked to abstain from alcohol, caffeine and exercise in the 24 hours before the testing. Cardiovascular fitness was assessed with a maximal incremental test on a stationary cycle ergometer (Excalibur sport bicycle ergometer, Lode, Netherlands) using a step protocol previously reported in young adults [31]. Participants were fitted with a heart rate strap (Polar T34, Polar Electro Oy, Finland) and facemask for the duration of the test. The protocol comprised a two-minute warm-up at 50 Watts, after which the load was increased by 25 Watts every 60 seconds until volitional exhaustion. Oxygen consumption (V̇O_2_) was measured continuously throughout the test for the determination of V̇O_2max_. The test was terminated when participants cycled below 60 revolutions per minute for 5 seconds, or the American College of Sports Medicine (ACSM) exercise termination criteria were met. V̇O_2max_ was verified by a stable V̇O_2_ despite an increased workload, a heart rate within 10% of the predicted maximum and a respiratory exchange ratio > 1.1.

Muscular strength was determined with a five repetition maximum (5RM) test of lower body (squat and split squat) and upper body (pectoral-fly and upright row) exercises using a standardised protocol according to the ACSM [32]. The upper and lower body exercises were chosen to activate multiple large muscle groups. Furthermore, a 5-RM assessment was selected for safety reasons and the untrained state of participants. From the 5-RM results, estimated 1-RM was calculated using the formula below [33]:


$$\begin{array}{l} \text{Upper body calculation}:1\text{-RM}=1.1307\left(5\text{-RM}\right)+0.6998.\\ \text{Lower body calculation}:1\text{-RM}=1.09703\left(5\text{-RM}\right)+14.2546. \end{array}$$

### Training Session

On the day of testing, participants reported to Deakin University at approximately 7.30 am after an overnight fast. Resting measurements of heart rate and oxygen saturation (SpO_2_; Prince 100F handheld pulse-oximeter with finger attachment, Healforce, China) were taken and a venous blood sample (10 mL) was collected from an antecubital vein. Participants then entered the environmental chamber for a 10-minute acclimation, followed by a five-minute low-intensity warm up on a cycle ergometer. The resistance exercise session consisted of four sets and ten repetitions of four exercises (in the following order); split squat, pectoral fly, standing row and squat. The exercises were conducted at 70% of the participants calculated 1-RM with a one-minute rest between sets and a two-minute rest between exercises. Upon completion of the final set, a 22-gauge intra-vascular catheter (Smiths Medical International) was inserted into an antecubital vein. Venous blood was collected immediately post-exercise and at 15, 30, 60, 120 and 180 minutes post-exercise. Blood was collected with a syringe at each time point and carefully transferred to the vacutainer to reduce the risk of haemolysis in the blood samples. Participants were provided a standardised meal for dinner that comprised of approximately 65% carbohydrates, 20% fat and 15% protein. Participants returned the following morning rested and fasted for a 24-hour blood sample.

SpO_2_ and heart rate were measured after the 10-minute acclimation to the environmental condition and immediately after each resistance exercise set was completed. Rating of perceived exertion (RPE) was assessed using the Borg Category Ratio 10 scale. Responses ranged from no exertion to maximal exertion on a 0–10 scale. Blood glucose and lactate were measured at each time point using an Accu-chek Performa Nano device (Roche Diabetes Care, Mannheim, Germany) and lactate pro 2 device (Arkay Factory Inc., Shiga, Japan), respectively.

A full blood count was performed on the EDTA-treated whole blood samples using a haematology analyser (DhX500, Beckman Coulter, Australia) to quantify total leukocyte, neutrophil, lymphocyte, monocyte, eosinophil and basophil concentration. The full blood count also quantified red blood cells, haemoglobin and platelets. Samples were run on the same day as blood collection. The haematology analyser was regularly cleaned and a 3-point calibration was performed before testing each sample batch. Due to the plasma volume shifts that occur with exercise, these were calculated and accounted for using the Dill and Costill formula [34].

### Statistical analysis

Data were graphed using GraphPad Prism (version 8.0.0) and tested for normality using the D’Agostino & Pearson test. Basophils did not pass the normality test and therefore underwent Log_10_ transformation prior to analysis. An independent samples T-test was used to check for differences between normoxia and hypoxia at baseline.

We used a restricted maximum likelihood linear mixed-effects model with fixed effects of group and time (and their interaction) and random intercepts for individuals (allowing for correlated measures across time points) using SPSS (IBM SPSS Statistics 26). This model was used to examine the interaction between group (normoxia, hypoxia) and time (Pre, post 0, 15, 30, 60, 120, 180 minutes and 24 hours). For the SpO_2_, heart rate and RPE values, there were six levels of time (Rest, acclimated rest, split squat, pectoral fly, standing row, squat). If a significant interaction was present (p < 0.05), pairwise comparisons were used to identify significant differences between groups at each time point.

## RESULTS

There were no significant differences in baseline characteristics between the experimental groups (independent samples T-test; p > 0.05).

**FIG. 1:**
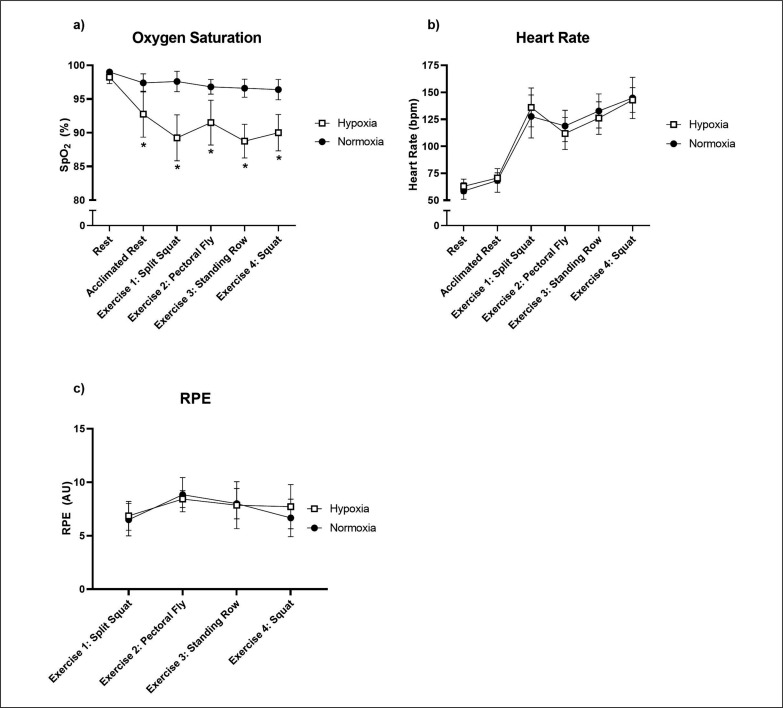
Oxygen saturation (a), heart rate (b) and rating of perceived exertion (RPE; c) responses to an acute bout of resistance exercise in normoxia or hypoxia. Mean ± SD. ^*^ Significant difference between hypoxia and normoxia (p < 0.05).

There was a significant time × group interaction for SpO_2_ (F[5,35.0] = 5.19, p = 0.001; Figure 1a). Pairwise comparisons showed that SpO_2_ was significantly lower in the hypoxic group following the 10-minute acclimation (estimated mean difference = -4.65 [95% CI: -2.11,3.61], p = 0.002), the split squat (estimated mean difference = -8.35 [95% CI: -11.21,-5.49], p < 0.001), pectoral fly (estimated mean difference = -5.30 [95% CI: -8.16,-2.44], p = 0.001), standing row (estimated mean difference = -7.85 [95% CI: -10.71,-4.99], p < 0.001) and squat exercise (estimated mean difference = -6.40 [95% CI: -9.26, -3.54], p < 0.001), compared to the normoxic group. There was no significant time × group interaction for heart rate (F[5,51.0] = 0.96, p = 0.453; Figure 1b), though there was a significant effect of time (F[5,51.0] = 103.59, p < 0.001), with heart rate peaking after the final squat exercise in both groups. RPE did not show a significant time × group interaction (F[3,33.0] = 0.82, p = 0.494), though there was a significant effect of time (F[3,33.0] = 5.83, p = 0.003; Figure 1c).

**FIG. 2:**
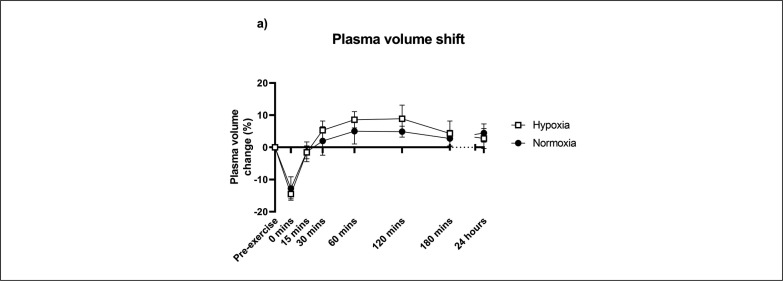
Plasma volume responses to an acute bout of resistance exercise in normoxia or hypoxia. Mean ± SD.

Plasma volume shift was accounted for in all blood analysis due to the known plasma shifts that occur with intense exercise between the systemic circulation and the extracellular space (Figure 2). The change in plasma volume was not different between hypoxic and normoxic groups (F[7,61.1] = 0.94, p = 0.482), though there was a significant effect of time (F[7,61.1] = 19.2, p < 0.001) where there was a decrease in plasma volume immediately post-exercise that returned close to baseline by 15 minutes.

**FIG. 3:**
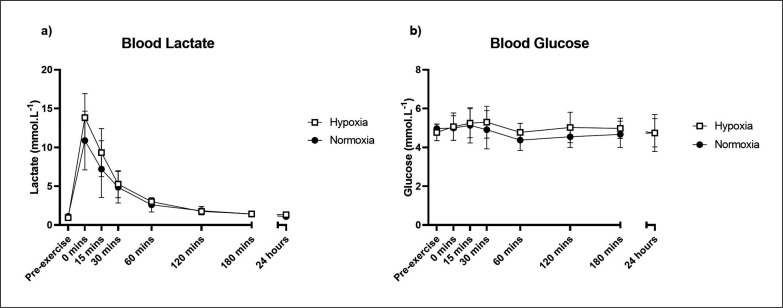
Blood lactate (a) and blood glucose (b) responses to an acute bout of resistance exercise in normoxia or hypoxia. Mean ± SD.

There was no significant time × group interaction for blood lactate (F[7,75.1] = 1.80, p = 0.100; Figure 3a). There was however a significant effect of time (F[7,61.1] = 88.56, p < 0.001), where both the normoxic and hypoxic groups showed a significant increase in blood lactate above the control group at 0, 15 and 30 minutes post-exercise. Blood glucose was not significantly different between groups (F[7,75.0] = 1.10, p = 0.370; Figure 3b), though there was a significant effect of time (F[7,75.1] = 3.30, p = 0.004).

**FIG. 4:**
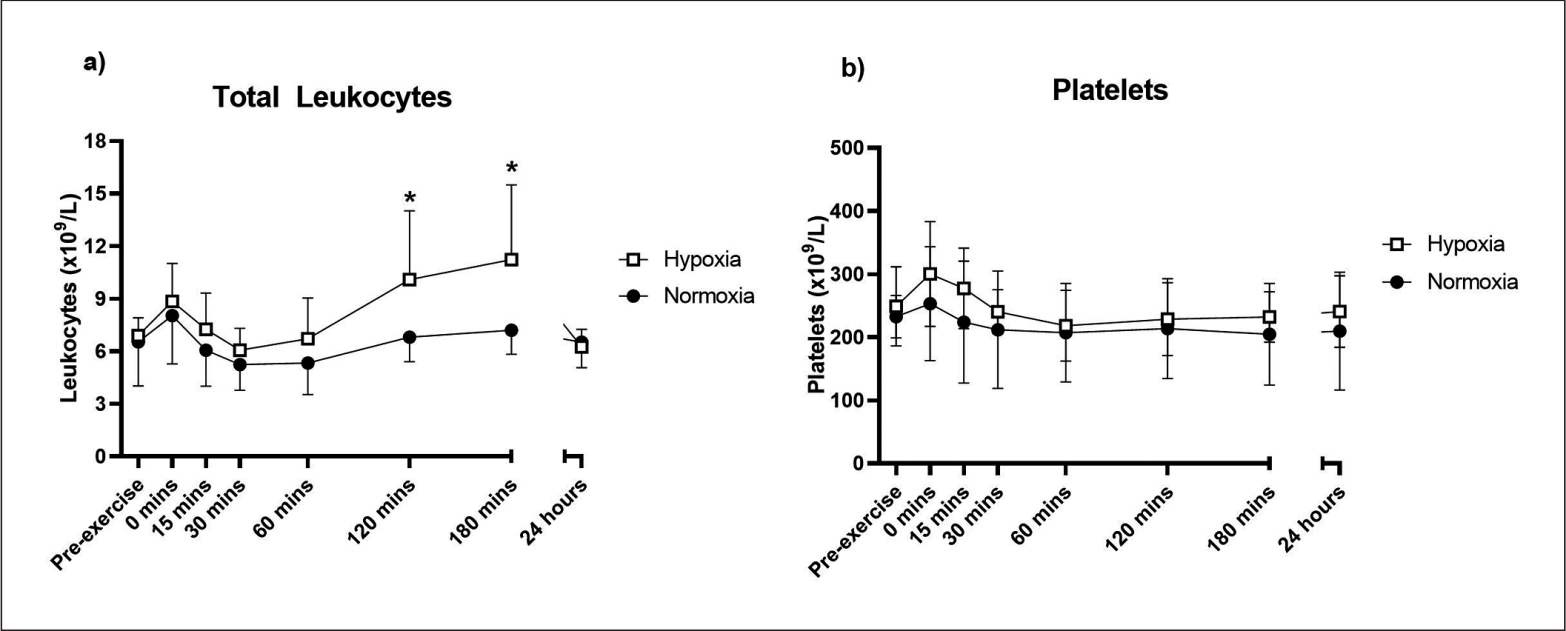
Total leukocyte (a) and platelet (b) responses to an acute bout of resistance exercise in normoxia or hypoxia. Mean ± SD. * Significant difference between hypoxia and normoxia (p < 0.05).

There was a significant time × group interaction for total leukocyte concentration in the 24 hours following the acute resistance exercise bout (F[7,75.1] = 4.43, p < 0.001; Figure 4a). Pairwise comparisons showed that total leukocyte concentration was significantly higher in hypoxia at 120 (estimated mean difference = 3.28 [95% CI: 0.67,5.90], p = 0.016) and 180 minutes (estimated mean difference = 3.89 [95% CI: 1.25,6.51], p = 0.006) post-exercise compared to normoxia. Platelets showed no significant time × group interaction (F[7,74.1] = 0.77, p = 0.615), though there was a significant effect of time (F[7,75.1] = 5.80, p < 0.001; Figure 4b).

**FIG. 5:**
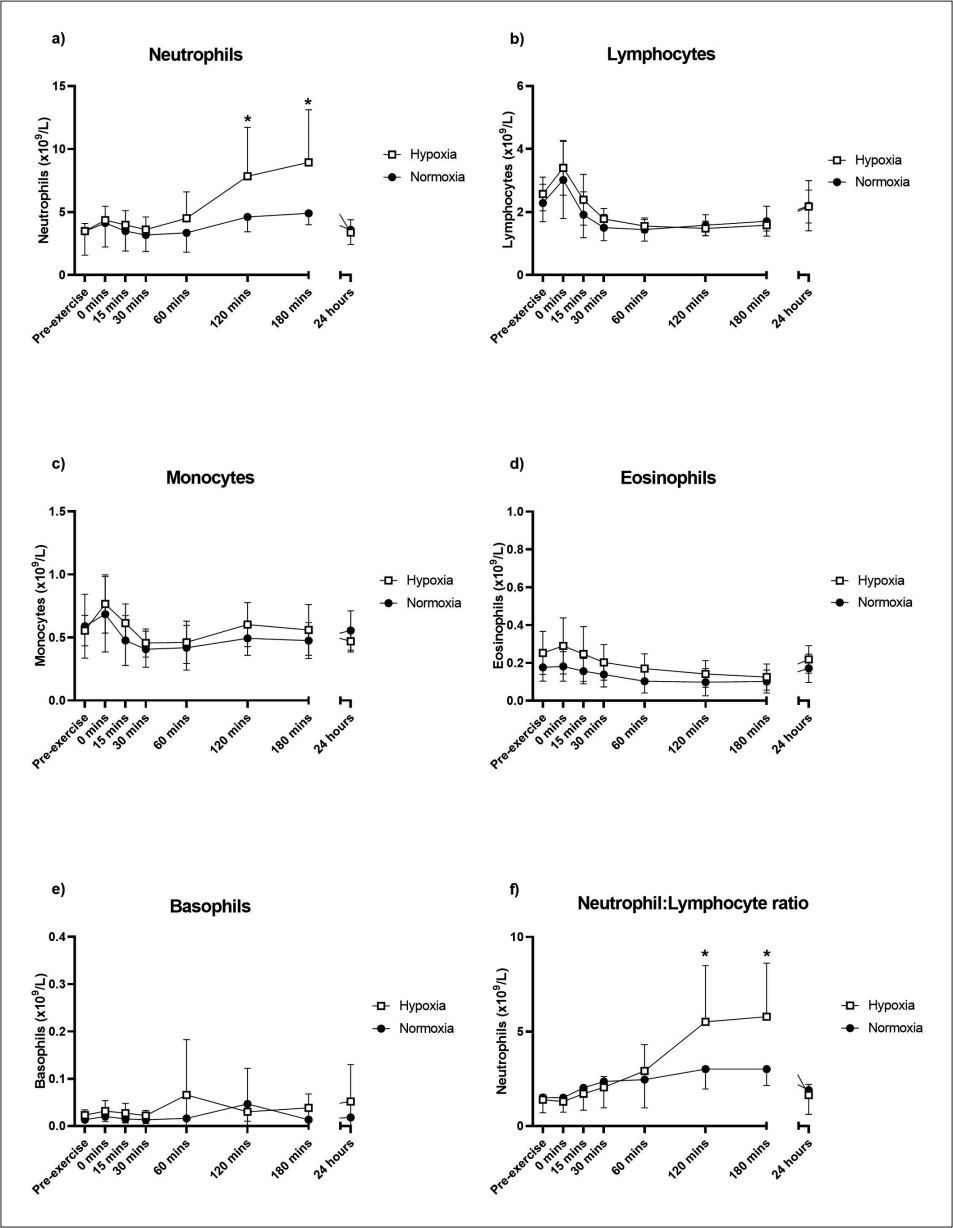
Neutrophil (a), lymphocyte (b), monocyte (c), eosinophil (d), basophil (e) and neutrophil/lymphocyte (f) ratio responses to an acute bout of resistance exercise in normoxia or hypoxia. Mean ± SD. * Significant difference between hypoxia and normoxia (p < 0.05).

There was a significant time × group interaction for neutrophil concentration in the 24 hours following the acute resistance exercise bout (F[7,75.1] = 5.22, p < 0.001; Figure 5a). Pairwise comparisons showed that neutrophils were significantly higher in hypoxia at 120 (estimated mean difference = 3.24 [95% CI: 1.04,5.43], p = 0.006) and 180 minutes (estimated mean difference = 3.90 [95% CI: 1.67,6.13], p = 0.001) post-exercise compared to normoxia.

Lymphocyte concentrations did not show a significant time × group interaction (F[7,75.1] = 0.98, p = 0.456; Figure 5b). However, there were a significant effect of time on lymphocyte numbers (F[7,75.1] = 25.12, p < 0.001). There was no significant time × group interaction present for monocytes (F[7,75.1] = 1.38, p = 0.227; Figure 5c), though there was a significant time effect (F[7,75.1] = 8.88, p < 0.001). There was no significant group × time interactions for basophils (F[7,80.0] = 0.57, p = 0.780) or eosinophils (F[7,75.0] = 1.02, p = 0.424), though eosinophils showed a significant effect of time (F[7,75.0] = 13.0, p < 0.001; Figure 5d, e). The neutrophil to lymphocyte ratio showed a significant time × group interaction (F[7,75.1] = 4.86, p < 0.001; Figure 5f). Pairwise comparisons showed that the ratio was significantly higher in hypoxia at 120 (estimated mean difference = 2.50 [95% CI: 0.96,4.04], p = 0.002) and 180 minutes (estimated mean difference = 2.70 [95% CI: 1.12,4.27], p = 0.001) post-exercise compared to normoxia.

## DISCUSSION

This study characterised the acute leukocyte responses of healthy adults to a single bout of resistance exercise in normobaric hypoxia. We hypothesised that hypoxia would amplify the leukocyte responses to acute resistance exercise.

For the first time, we showed that performing resistance exercise in normobaric hypoxia significantly increases neutrophil concentrations at 120 minutes and 180 minutes post-exercise compared to normoxia. Although the underlying cause of the neutrocytosis is unknown, changes in growth hormone are of great interest in the hypoxic training literature. Exposure to hypoxia during an acute bout of resistance exercise increases the growth hormone response up to 60 minutes post-exercise [28, 29]. Intravenous injection of 2IU recombinant human growth hormone can induce a significant neutrocytosis after 120 minutes [35]. Leukocyte subpopulations including neutrophils possess growth hormone receptors [36] and type 1 IGF-1 receptors [37]. Therefore, it is plausible that the amplified growth hormone response to resistance exercise observed in previous hypoxic studies [28, 29] is linked to the neutrocytosis observed in the current study. However, this is speculative and further research is needed to confirm this potential relationship. Currently the functional consequences of the neutrocytosis are unclear, though it could help to respond to invading pathogens in the circulation [38] and support the repair of skeletal muscle following damaging resistance exercise [39]. Further research is needed to examine the functional capacity of neutrophils following resistance exercise in hypoxia. The neutrophil to lymphocyte ratio was also elevated in the hypoxic group at 120 and 180 minutes post-exercise, though it is currently unclear if there are any functional benefits or consequences from an acute increase in this ratio. The neutrophil to lymphocyte ratio is typically measured at rest as prognostic biomarker for conditions such as cancer [40] and sepsis [41].

Surprisingly, there was no effect of hypoxia on circulating lymphocytes in the 24 hours following resistance exercise. Both the normoxic and hypoxic trained groups showed the classic bi-phasic response [42], where a significant increase in lymphocytes at 0 minutes post-exercise was followed by suppression below baseline (lymphocytopenia) for up to three hours. The exercise immunology field is still divided on whether exercise-induced lymphocytopenia increases the risk of infection and illness as there is little causal data available [43]. Importantly, these findings suggest that performing moderate-intensity resistance exercise under hypoxic conditions does not alter post-exercise immunity. Further studies are needed to determine if leukocyte function changes in response to resistance training in hypoxia. In particular, chronic training programs in hypoxia with larger sample sizes may be able to detect more subtle and long-term leukocyte responses to hypoxic resistance training.

SpO_2_ levels were significantly but safely reduced during the resistance training bout in hypoxia, with the SpO_2_ in the hypoxic group dropping to a minimum of 91.8 ± 4.7% following the split squat compared to 98.0 ± 1.7% in normoxia. These SpO_2_ levels were similar to other resistance training protocols in similar hypoxic conditions [17]. There was no difference in RPE between the hypoxic and normoxic groups, suggesting that the difficulty of hypoxic exercise was not greater than normoxic exercise. After performing the exercise session, 10 out of the 13 participants thought that they were randomised to the hypoxic group, suggesting that the participants were successfully blinded to their environmental condition.

Generally speaking, hypoxia is thought to elicit a greater relative exercise intensity than normoxia [22, 23], which in turn increases metabolic stress, activates sympathetic pathways and induces a greater immune response [21]. However, in the present study, blood glucose, blood lactate and heart rate were not significantly different between experimental groups, despite evidence that acute normobaric hypoxia enhances glucose disposal into skeletal muscle through contraction-mediated pathways [44]. Furthermore, the blood lactate result was not consistent with most other resistance training protocols where lactate is significantly elevated following hypoxic exercise [45, 46]. It is possible that the high training intensity and load used in our study elicited a near-maximal blood lactate response and therefore the additional stimulus of hypoxia was marginal. Blood lactate peaked at approximately 14 mmol/L^-1^ in hypoxia immediately post-exercise, compared to peaks of approximately 6–9 mmol/L^-1^ in hypoxic training studies that used a similar exercise intensity [28, 47, 48]. Therefore, relative exercise intensity may not have significantly contributed to the immune response in this study. The immune response to hypoxic resistance exercise is likely multifaceted, with possible contributions from other hormonal factors (Eg. growth hormone) and the activation of immune-related pathways such as HIF-1 and NFκB.

## CONCLUSIONS

Exposure to normobaric hypoxia during an acute bout of resistance exercise increases the neutrophil response at 120 and 180 minutes post-exercise compared to normoxia. Further research is required to examine the causes and functional implications of the hypoxia-induced neutrocytosis. No other leukocyte populations were affected by hypoxic resistance exercise, suggesting that in healthy males, the addition of hypoxia during resistance training may allow positive training adaptations without negatively impacting immune function. However, caution should be applied when using more severe hypoxic stimuli and longer hypoxic exposure times than the current study, as further investigation is needed to assess these chronic impacts. Similarly, the long-term effects on leukocytes must be determined to conclusively state that hypoxia is a safe and effective tool for exercise prescription.
